# Risk management actions following genetic testing in the Cancer Health Assessments Reaching Many (CHARM) Study: A prospective cohort study

**DOI:** 10.1002/cam4.6485

**Published:** 2023-08-30

**Authors:** Boya Guo, Sarah Knerr, Tia L. Kauffman, Kathleen F. Mittendorf, Erin Keast, Marian J. Gilmore, Heather Spencer Feigelson, Frances L. Lynch, Kristin R. Muessig, Sonia Okuyama, Jamilyn M. Zepp, David L. Veenstra, Li Hsu, Amanda I. Phipps, Sara Lindström, Michael C. Leo, Katrina A. B. Goddard, Benjamin S. Wilfond, Beth Devine, the CHARM Study team, Jake Allen, Laura M. Amendola, Katherine P. Anderson, Frank Angelo, Briana L. Arnold, Cecelia Bellcross, Tiffany Bendelow, Barbara B. Biesecker, Kristin D. Breslin, Joanna E. Bulkley, Kristina F. Booker, Mikaella Caruncho, James V. Davis, Sonia Deutsch, Michael O. Dorschner, Devan M. Duenas, Donna J. Eubanks, Amanda S. Freed, Marian J. Gilmore, Clay Greaney, Inga Gruß, Claudia Guerra, Joan Holup, Jessica Ezzell Hunter, Chalinya L. Ingphakorn, Paige Jackson, Gail P. Jarvik, Charisma L. Jenkins, Galen Joseph, Leah S. Karliner, Alyssa H. Koomas, Stephanie A. Kraft, Mi H. Lee, Robin Lee, Sandra Soo‐Jin Lee, Hannah S. Lewis, Elizabeth G. Liles, Nangel M. Lindberg, Carmit K. McMullen, Elizabeth Medina, Kristin R. Muessig, Sonia Okuyama, C. Samuel Peterson, Angela R. Paolucci, Rosse Rodriguez Perez, Kathryn M. Porter, Chelese L. Ransom, Ana Reyes, Leslie S. Riddle, Sperry Robinson, Bradley A. Rolf, Alan F. Rope, Emily Schield, Jennifer L. Schneider, Kelly J. Shipman, Brian H Shirts, Elizabeth Shuster, Sapna Syngal, Britta N. Torgrimson‐Ojerio, Chinedu Ukaegbu, Meredith L. Vandermeer, Alexandra M. Varga, David L. Veenstra, W. Chris Whitebirch, Larissa Lee White

**Affiliations:** ^1^ School of Public Health University of Washington Seattle Washington USA; ^2^ Center for Health Research, Kaiser Permanente Northwest Portland Oregon USA; ^3^ Vanderbilt‐Ingram Cancer Center Vanderbilt University Medical Center Nashville Tennessee USA; ^4^ Department of Translational and Applied Genomics Center for Health Research Portland Oregon USA; ^5^ Institute for Health Research Denver Colorado USA; ^6^ Division of Oncology, Denver Health and Hospital Authority Denver Colorado USA; ^7^ The Comparative Health Outcomes, Policy, and Economics (CHOICE) Institute, School of Pharmacy University of Washington Seattle Washington USA; ^8^ Division of Public Health Sciences Fred Hutchinson Cancer Center Seattle Washington USA; ^9^ Treuman Katz Center for Pediatric Bioethics Seattle Children's Research Institute Seattle Washington USA; ^10^ Department of Pediatrics, Division of Bioethics and Palliative Care University of Washington Seattle Washington USA

**Keywords:** genetic testing, genomic sequencing, health service utilization, hereditary breast and ovarian cancer, hereditary cancer, Lynch syndrome, underserved populations

## Abstract

**Background:**

Genetic testing can identify cancer risk early, enabling prevention and early detection. We describe use of risk management interventions following genetic testing in the Cancer Health Assessment Reaching Many (CHARM) study. CHARM assessed risk and provided genetic testing to low income, low literacy, and other underserved populations that historically face barriers to accessing cancer genetic services.

**Methods:**

CHARM was implemented in Kaiser Permanente Northwest (KPNW) and Denver Health (DH) between 2018 and 2020. We identified post‐testing screening (mammography, breast MRI, colonoscopy) and surgical (mastectomy, oophorectomy) procedures using electronic health records. We examined utilization in participants who did and did not receive actionable risk management recommendations from study genetic counselors following national guidelines.

**Results:**

CHARM participants were followed for an average of 15.4 months (range: 0.4–27.8 months) after results disclosure. Less than 2% (11/680) received actionable risk management recommendations (i.e., could be completed in the initial years following testing) based on their test result. Among those who received actionable recommendations, risk management utilization was moderate (54.5%, 6/11 completed any procedure) and varied by procedure (mammogram: 0/3; MRI: 2/4; colonoscopy: 4/5; mastectomy: 1/5; oophorectomy: 0/3). Cancer screening and surgery procedures were rare in participants without actionable recommendations.

**Conclusion:**

Though the number of participants who received actionable risk management recommendations was small, our results suggest that implementing CHARM's risk assessment and testing model increased access to evidence‐based genetic services and provided opportunities for patients to engage in recommended preventive care, without encouraging risk management overuse.

## 1 INTRODUCTION

Hereditary breast and ovarian cancer syndrome (HBOC) and Lynch syndrome (LS) account for a significant proportion of inherited malignancies^1^, ^2^, ^3^, ^4^, ^5^ and have guidelines supporting their early detection through predictive genetic testing.^6^, ^7^, ^8^ Multi‐gene panel testing through next‐generation sequencing is thus increasingly used to identify individuals at high risk of hereditary cancers with a goal of enabling early screening (e.g., mammography, breast MRI) and risk reduction (e.g., mastectomy, chemoprevention) measures.

There are marked disparities in delivering genetic counseling and genetic testing services across sociodemographic groups.^9^, ^10^, ^11^, ^12^, ^13^, ^14^ The Cancer Health Assessment Reaching Many (CHARM) study developed and tested a multimodal intervention designed to increase access to clinically indicated genetic testing and to narrow inequities in cancer genetic service delivery.^15^ Specifically, CHARM piloted a hereditary cancer risk assessment program in Kaiser Permanente Northwest (KPNW), an integrated health care delivery system in Oregon and southwest Washington state, and Denver Health (DH), an integrated safety‐net health system in Denver County, Colorado.^16^ Patient recruitment prioritized outreach to groups with historically limited access to cancer genetic testing, including low income, low literacy, and minoritized racial and ethnic groups, as well as individuals with limited or unknown family history.

Prior studies of patient's risk management use in initiatives designed to increase access to genetic testing in healthy populations (i.e., not in the context of a disease diagnosis) have largely shown moderate to high engagement with subsequent recommended care.^17^, ^18^, ^19^, ^20^, ^21^ However, with a few notable exceptions, these initiatives have engaged “early test adopters” and we have limited understanding of healthcare utilization patterns following cancer genetic testing in populations who traditionally face barriers in accessing genetic servies.^13^, ^22^ Whether returning genetic test results with no clear risk‐management implications, for instance variants of uncertain significance (VUS), leads to unnecessary risk management utilization due to patient anxiety or clinician mismanagement is another question of interest, particularly given health care resource constraints these populations already experience.^14^, ^23^, ^24^

To increase data about post‐testing risk management behavior in populations known to have access barriers, this paper describes risk management actions taken by CHARM study participants who did and did not receive actionable risk management recommendations (based on their genetic test results and age) from study genetic counselors.

## 2 METHODS

### 2.1 CHARM overview

The CHARM study was one of seven projects in the National Institutes of Health's Clinical Sequencing Evidence‐Generating Research (CSER) consortium (cser‐consortium.org).^25^ The recruitment process and study design of the CHARM study have been described in detail elsewhere.^15^

Briefly, English‐ and Spanish‐speaking patients aged 18–49 years from KPNW and DH were invited to complete an online family history risk assessment survey between August 2018 and March 2020. Invitations included email, text message, post‐card, and phone outreach as well as in person tabling in both systems. Eligible participants were at risk of HBOC or LS as assessed by the Breast Cancer Genetics Referral Screening Tool (B‐RST 3.0)^26^ (HBOC syndrome) or Prediction Model for Gene Mutations (PREMM_5_)^27^ (LS). Patients were also eligible to participate in the study if they had limited family knowledge (unknown family history) or structure (less than two female members living beyond age 45 on either side).^28^ Eligible patients were presented with an online education and consent application, including the option to receive additional findings.^29^ Participants received exome‐based panel testing through a CLIA‐certified laboratory at the University of Washington.^15^ Variants were annotated in 39 genes associated with hereditary cancer risk and 77 noncancer, medically actionable secondary finding genes.^16^ Genetic testing results were classified as either pathogenic/likely pathogenic (P/LP) variant(s); negative (benign and likely benign variants); or only variants of uncertain significance (VUS). Genetic testing was provided at no cost to CHARM participants.

Board‐certified study genetic counselors provided risk management recommendations based on the most recent National Comprehensive Cancer Network (NCCN) guidelines on cancer risk management at the time of test result disclosure, which were documented in participants' electronic health records (EHR) following standard practice in the two systems (Table S1).^7^, ^8^ Any downstream medical care recommended because of the test was considered part of usual care and subject to the participant's medical benefits or coverage through established financial assistance programs in the two health systems. For participants who received P/LP test results and those with negative or VUS but who were at clinically significant risk for hereditary cancers based on their family history, results were disclosed over the phone. For participants who qualified on the basis of limited family structure/knowledge and received negative results, results were returned by letter, provided in English or Spanish based on patient's preference for risk assessment and consent.

### 2.2 Cohort study design

We included eligible patients who consented for the CHARM study participation and excluded those who declined or were lost to follow‐up. We further excluded CHARM participants who had an inadequate exome sequencing sample or died or disenrolled from health system before result disclosure. We followed study members to determine use of screening modalities and risk reducing surgeries from the time when a participant received their genetic test results to the end of study follow‐up (March 31, 2021 for KPNW and February 28, 2021 for DH), health plan disenrollment, or death. For each risk management procedure (mammography, breast MRI, colonoscopy, risk‐reducing mastectomy **(**RRM), and risk‐reducing salpingo‐oophorectomy (RRSO)), we excluded individuals without relevant organ(s) at study entry. With the exception of colonoscopy, we also excluded individuals who did not report their sex at birth as female (Table S2) on the family cancer history screening tool.

### 2.3 Demographic and clinical data

KPNW and DH maintain comprehensive administrative and clinical databases that are available for research. Data from the EHR, administrative systems, and external claims are incorporated into a research data warehouse using a unique health record number for each health plan member.^30^, ^31^ We searched data for evidence of surgery (RRM and RRSO) and cancer screening (mammography, breast MRI, and colonoscopy) following results disclosure using procedure codes listed in Table S3. Participant characteristics were obtained from the CHARM study tracking system, which contained EHR‐derived and self‐reported data from the web‐based patient facing survey, including sex assigned at birth, age, race and ethnicity, insurance status, education, household income, CHARM risk assessment results, and CHARM genetic testing results. Self‐reported race and ethnicity data was primarily extracted from the survey. For patients without self‐reported race and ethnicity data from survey, data from EHR was used. At KPNW, incident cancer diagnoses were determined using the tumor registry, whereas at DH, they were determined by review of patient encounters with oncology, surgery, women's care, and acute care services (Table S4). The health plan membership data was used to ascertain death dates.

### 2.4 Statistical analysis

We described study participants overall, by study site, and by membership in sociodemographic groups traditionally excluded from genomic medicine efforts. Following definitions developed by the CSER consortium, we classified individuals with historically limited access to cancer genetic testing as anyone who: (1) self‐reported Hispanic or Latino ethnicity and/or a race other than White, (2) resided in a medically underserved area (>20% of households are below the poverty level and/or > 20% have less than a high school education) determined using geocoded census tract information, (3) completed any study survey or risk assessment tool in Spanish, (4) had less than high school education, (5) had income <200% federal poverty level or Medicaid insurance, or (6) was uninsured.

We examined risk management uptake in two groups: those who did and did not receive actionable risk management recommendations (i.e., procedures that could be completed in the initial years after genetic results disclosure) from CHARM study genetic counselors per current NCCN guidelines (Table S1). In those with actionable recommendations, we described risk management use within individual participants and summarized utilization by procedure. For each procedure, we categorized participants into two mutually exclusive groups: (1) unaffected individuals (*no personal history of the relevant cancer*), and (2) cancer survivors (*personal history of the relevant cancer*). In those without actionable recommendations, we summarized utilization by procedure and categorized participants into four mutually exclusive groups: (1) not yet recommended age (*had a P/LP in a relevant gene, but did not reach the recommended age to start that cancer risk management procedure*), (2) other P/LP (*received a P/LP result in a gene not relevant to that cancer risk management procedure*), (3) VUS only (*received a VUS result only*), and (4) negative (*received a negative result)*. For each procedure, we reported the number of individuals who had at least one corresponding code after result disclosure and calculated the mean and median time between results disclosure and the initial uptake of that procedure. In an exploratory analysis, we examined overall uptake of risk management (any screening or surgery) by personal cancer history (prior breast, ovarian, or colorectal cancer vs. none), health system (KPNW vs. DH), membership in a group with known access barriers (yes vs. no), and family history knowledge or structure (known vs unknown) in those without actionable recommendations.

For all analyses, categorical variables were described using frequencies and percentages, and continuous variables were reported as means, standard deviations (SD), medians, and interquartile ranges. Two‐tailed chi‐square or Fisher's exact tests were used to compare categorical variables. Two‐sample t‐tests were used to compare continuous variables. A *P*‐value <0.05 was considered statistically significant. Analyses were conducted using R version 4.2.1.

## 3 RESULTS

### 3.1 Participant characteristics

**FIGURE 1:**
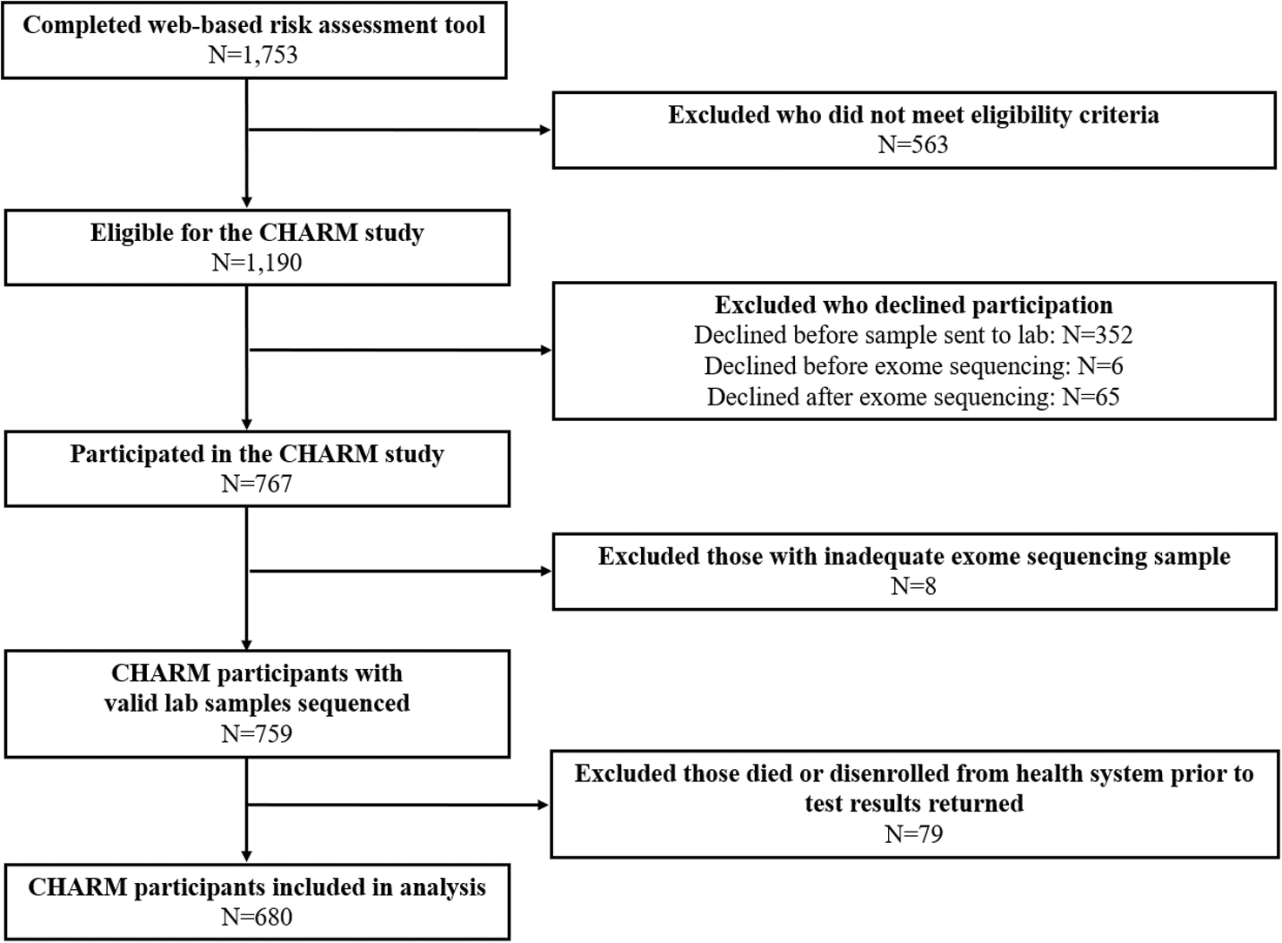
CHARM study participation flow chart.

A total of 680 CHARM participants were included in the final analysis (Figure 1). Of these participants, 27.6% were recruited from DH and 72.4% were recruited from KPNW. Overall, 70.0% belonged to a group traditionally excluded from genomic medicine efforts (Table 1). The average age at the time of family cancer history online risk assessment completion was 36.4 years old (SD = 8.2, range: 18.0–49.0). The average time between risk assessment completion and result disclosure was 4 months (SD = 1.5, range: 1.3–13.5) and the average time from return disclosure to end of study follow‐up was 15.4 months (SD = 6.0, range: 0.4–27.8). Almost one‐half of participants were non‐Hispanic White (46.5%, *n* = 316), assigned female sex at birth (79.7%, *n* = 542), and were commercially or privately insured (60.0%, *n* = 408). About 20% had a high school education or less and about 40% (*n* = 281) reported income <200% federal poverty level. Thirty (4.4%) participants had a personal history of breast, ovarian, or colorectal cancer at study entry. Participant characteristics differed by study site. Compared to KPNW, DH participants were more often assigned female sex at birth, belonged to a minoritized racial or ethnic group, were publicly insured or uninsured, had less than high school education, and/or had low income.

**TABLE 1:** Demographic and clinical characteristics of CHARM participants.

Characteristics	Total, *N* (%)	Study site, *n* (%)			Access barriers^a^, *n* (%)		
	Total *N* = 680	DH *N* = 188	KPNW *N* = 492	*p* value	Yes *N* = 476	No *N* = 204	*p* value
Sex assigned at birth							
Female	542 (79.7)	169 (89.9)	373 (75.8)	**<0.001**	398 (83.6)	144 (70.6)	**<0.001**
Male	138 (20.3)	19 (10.1)	119 (24.2)		78 (16.4)	60 (29.6)	
Age at risk assessment, year							
Mean (SD)	36.4 (8.2)	36.7 (7.9)	36.3 (8.3)	0.555	36.2 (8.4)	36.9 (7.6)	0.319
Age at result returned, year							
Mean (SD)	36.8 (8.2)	37.0 (7.9)	36.7 (8.3)	0.621	36.5 (8.4)	37.3 (7.5)	0.273
Follow‐up time, month							
Mean (SD)	15.4 (6.0)	15.8 (4.4)	15.3 (6.5)	0.335	16.5 (6.7)	14.9 (5.6)	**0.002**
Race and ethnicity							
Asian	34 (5.0)	4 (2.1)	30 (6.1)	**<0.001**	34 (7.1)	0 (0)	**<0.001**
Black	37 (5.4)	5 (2.7)	32 (6.5)		37 (7.8)	0 (0)	
Hispanic or Latino	211 (31.0)	106 (56.4)	105 (21.3)		211 (44.3)	0 (0)	
White	316 (46.5)	54 (28.7)	262 (53.3)		113 (23.7)	203 (99.5)	
All other^b^	79 (11.6)	18 (9.6)	61 (12.4)		79 (16.6)	0 (0)	
Unknown	3 (0.4)	1 (0.5)	2 (0.4)		2 (0.4)	1 (0.5)	
Year of receiving test results							
2018	9 (1.3)	0 (0)	9 (1.8)	0.140	2 (0.4)	7 (3.4)	**<0.0001**
2019	361 (53.1)	105 (55.9)	256 (52.0)		236 (49.6)	125 (61.3)	
2020	310 (45.6)	83 (44.2)	227 (46.1)		238 (50.0)	72 (35.3)	
Insurance type							
Commercial or private	408 (60.0)	55 (29.7)	353 (72.2)	**<0.001**	224 (47.6)	184 (90.6)	**<0.0001**
Medicare	31 (4.6)	4 (2.2)	27 (5.5)		31 (6.6)	0 (0)	
Medicaid	96 (14.1)	61 (33.0)	35 (7.2)		96 (20.4)	0 (0)	
Uninsured	63 (9.3)	46 (24.9)	17 (3.5)		58 (12.3)	5 (2.5)	
Other/Unknown	82 (12.1)	4 (42.2)	14 (2.9)		21 (4.5)	1 (0.5)	
Education level							
Less than high school	41 (6.0)	34 (18.1)	7 (1.6)	**<0.001**	41 (8.6)	0 (0)	**<0.001**
High school	94 (13.8)	43 (22.9)	51 (11.4)		84 (17.7)	10 (4.9)	
Post‐high school or associate degree	132 (19.4)	27 (14.4)	105 (23.5)		97 (20.4)	35 (17.2)	
Bachelor's degree	246 (36.2)	49 (26.1)	197 (44.2)		158 (33.2)	88 (43.1)	
Graduate or professional	108 (15.9)	22 (11.7)	86 (19.3)		50 (10.5)	58 (28.4)	
Unknown/did not respond	59 (8.7)	13 (6.9)	46 (9.4)		46 (9.7)	13 (6.4)	
Low‐income (<200% FPL or Medicaid)							
Yes	281 (41.3)	137 (72.9)	144 (29.3)	**<0.0001**	281 (59.0)	0 (0)	**<0.001**
No	399 (58.7)	51 (27.1)	348 (70.7)		195 (41.0)	204 (100.0)	
Hereditary cancer history^c^							
Yes	30 (4.4)	12 (6.4)	18 (3.7)	0.122	25 (5.3)	5 (2.5)	0.103
No	650 (95.6)	176 (93.6)	474 (96.3)		451 (94.8)	199 (97.6)	
Baseline risk assessment tool used to determine eligibility							
PREMM_5_	71 (10.4)	15 (8.0)	56 (11.4)	**0.008**	54 (11.3)	17 (8.3)	0.271
B‐RST	383 (56.3)	125 (66.5)	258 (52.4)		274 (57.6)	109 (53.4)	
PREMM_5_ and B‐RST	79 (11.6)	20 (10.6)	59 (12.0)		51 (10.7)	28 (13.7)	
Limited family history/knowledge	147 (21.6)	28 (14.9)	119 (24.2)		97 (20.4)	50 (24.5)	
Genetic testing result							
P/LP							
Hereditary cancer related	23 (3.4)	18 (3.7)	5 (2.7)	0.896	10 (2.1)	13 (6.4)	**<0.001**
Other	70 (10.3)	49 (10.0)	21 (11.2)		38 (8.0)	32 (15.7)	
VUS only	48 (7.1)	35 (7.1)	13 (6.9)		36 (7.6)	12 (5.9)	
Negative	539 (79.3)	390 (79.3)	149 (79.3)		392 (82.4)	147 (72.1)	

*Note*: The bold front indicates *p*‐values with statistical significance. Abbreviations: B‐RST, Breast Cancer Genetics Referral Screening Tool; DH, Denver Health; FPL, federal poverty level; KPNW, Kaiser Permanente Northwest; P/LP, pathogenic/likely pathogenic; PREMM_5_, Prediction Model for Gene Mutations; VUS, variant of uncertain significance.

^a^ Any of the following: (1) self‐reported Hispanic ethnicity and/or a race other than White, (2) reside in a medically underserved area (>20% of households are below the poverty level and/or > 20% have less than a high school education) determined using geocoded census tract information, (3) completed any study survey or risk assessment tool in Spanish, (4) had less than high school education, (5) had income <200% federal poverty level or Medicaid insurance, or (6) were uninsured.

^b^ All other: individuals identified in the medical record as Native American, Native Hawaiian or Pacific Islander, North African, Mediterranean, more than one race or ethnicity, or other (no further description given).

^c^ Hereditary cancer history: breast, ovarian, or colorectal cancer diagnosis before the risk assessment completion date.

### 3.2 Genetic testing results and risk management recommendations

In total, 93 out of 680 participants (13.7%) had at least one P/LP variant, 48 (7.1%) had a VUS only, and 539 (79.3%) had a negative result (Table 1). Of the 93 participants who had a P/LP result returned, 23 (24.7%) had a P/LP variant in a well‐established colorectal, breast, and/or ovarian cancer gene (Table S1). Among these 23 individuals, 11 received at least one actionable risk management recommendation from study genetic counselors. The remainder (*n* = 12) had no actionable risk management recommendation because they either completed surgery removing the organ(s) of interest (breast, ovaries, or colon) prior to receiving test results through CHARM or were too young to begin recommended risk management.

### 3.3 Screening and surgery in those with risk management recommendations

Use of risk management procedures in those with actionable recommendations from study genetic counselors is shown in Table 2. Over one‐half of participants with actionable recommendations pursued at least one risk management procedure during follow‐up (54.5%, 6/11). Of the five individuals who did pursue risk management, one had less than a month of observed follow‐up time and the remainder were quite young (23, 26, and 38 years old) when results were returned, particularly given the specific risk management procedures they were considering (Table 2). Examining use of recommended risk management by procedure, no participants (0/3) completed mammography, 50.0% (2/4) completed breast MRI, 80.0% (4/5) completed colonoscopy, 20.0% completed RRM (1/5), and none completed RRSO (0/3) during study follow‐up. Across procedures, cancer survivors had higher uptake rates than unaffected individuals and also completed risk management procedures sooner after results disclosure (Table 3).

**TABLE 2:** Characteristics of participants who received actionable recommendations from CHARM study genetic counselors, by study site.

Age at return, years	Sex assigned at birth	Race and ethnicity	Gene	Personal Cancer History	Had mammog‐ram	Had MRI	Had colonosc‐opy	Had RRM	Had RRSO	Follow‐up time, months
KPNW										
43	Female	Hispanic or Latino	*BRCA1*	Breast	No	Yes		Yes	No	9.17
26	Female	White	*BRCA2*	No		No		No		10.41
38	Female	Asian	*BRCA2*	Breast^a^					No	12.00
42	Female	White	*BRCA2*	No	No	Yes		No	No	11.07
45	Female	White	*CHEK2*	No	No	No	No			0.36
44	Female	White	*MSH6*	No			Yes			21.85
28	Female	White	*PALB2*	No				No		13.63
35	Female	White	*PMS2*	No			Yes			16.13
43	Female	White	*PMS2*	Colon			Yes			12.94
DH										
23	Female	White	*BRCA1*	No				No		16.03
31	Male	Asian	*MSH2*	No			Yes			11.93

Abbreviations: DH, Denver Health; KPNW, Kaiser Permanente Northwest; MRI, magnetic resonance imaging; RRM, risk‐reducing mastectomy; RRSO, risk‐reducing salpingo‐oophorectomy.

^a^ Had RRM prior to learning CHARM test results.

**TABLE 3:** Use of risk management after genetic test result disclosure in those who received actionable recommendations from CHARM study genetic counselors, by procedure.

	Total	Uptake	Time from result disclosure to procedure, weeks	
	*n*	*n* (%)	Mean (SD)	Median (IQR)
Mammography	3	0 (0)		
Unaffected^a^	2	0 (0)	NA	NA
Breast cancer survivor^b^	1	0 (0)	NA	NA
Breast MRI	4	2 (50.0)		
Unaffected^a^	3	1 (33.3)	4.4 (NA)	4.4 (NA)
Breast cancer survivor^b^	1	1 (100.0)	0.9 (NA)	0.9 (NA)
Colonoscopy	5	4 (80)		
Unaffected^a^	4	3 (75.0)	31.6 (31.7)	17.0 (13.4–42.5)
Colon cancer survivor^b^	1	1 (100.0)	0.1 (NA)	0.1 (NA)
RRM	5	1 (20.0)		
Unaffected^a^	4	0 (0)	NA	NA
Breast cancer survivor^b^	1	1 (100.0)	5.9 (NA)	5.9 (NA)
RRSO	3	0 (0)		
Unaffected^a^	3	0 (0)	NA	NA
Ovarian cancer survivor^b^	0	NA	NA	NA

Abbreviations: MRI, magnetic resonance imaging; RRM, risk‐reducing mastectomy; RRSO, risk‐reducing salpingo‐oophorectomy.

^a^ Individuals with P/LP variants in high‐risk genes, intact organ(s), no relevant personal cancer history, and reached recommended age.

^b^ Individuals with P/LP results in high‐risk genes, intact organ(s), relevant personal cancer history, and reached recommended age.

### 3.4 Screening and surgery in those without recommendations

Examining use of risk management procedures among participants who did not receive actionable recommendations from study genetic counselors, 24.2% (124/513) had mammography, 1.8% (9/512) had breast MRI, 5.1% (34/666) had colonoscopy, <1% (2/511) had RRM, and <1% (2/516) had RRSO during study follow‐up. Across procedures, uptake rates varied by genetic test results with no clear patterns. Both individuals who had RRM (one tested negative, one with a VUS) were breast cancer survivors (Table 4). The proportion of participants without actionable recommendations who used one or more risk management procedure during study follow‐up was similar between study sites, genetic testing access barriers (based on participant's sociodemographic profiles), and personal history of breast, ovarian, or colorectal cancer. Those with known family history had a higher uptake of one or more risk management procedures during study follow‐up compared to those with limited knowledge of their family history or structure (Table 5).

**TABLE 4:** Use of risk management after test result disclosure in those who did not receive actionable recommendations from CHARM study genetic counselors, by procedure.

	Total	Uptake	Time from result disclosure to procedure, weeks	
	*n*	*n* (%)	Mean (SD)	Median (IQR)
Mammography	513	124 (24.2)		
Not yet recommended age^a^	6	0 (0)	NA	NA
P/LP other^b^	57	17^e^ (29.8)	35.0 (28.0)	28.0 (17.4–42.7)
VUS only^c^	40	12^e^ (30.0)	37.0 (30.5)	30.3 (19.5–48.4)
Negative^d^	410	95^f^ (23.2)	35.6 (23.9)	31.0 (18.4–46.7)
Breast MRI	512	9 (1.8)		
Not yet recommended age^a^	5	1 (20.0)	37.3 (NA)	37.3 (NA)
P/LP other^b^	57	1 (1.8)	22.0 (NA)	22.0 (NA)
VUS only^c^	40	1 (2.5)	15.7 (NA)	15.7 (NA)
Negative^d^	410	6 (1.5)	34.5 (31.4)	29.4 (17.0–40.1)
Colonoscopy	666	34 (5.1)		
Not yet recommended age^a^	5	0 (0)	NA	NA
P/LP other^b^	81	5 (6.2)	36.8 (42.9)	21.2 (6.3–43.0)
VUS only^c^	47	5 (10.6)	40.7 (28.2)	29.4 (18.7–56.0)
Negative^d^	533	24 (4.5)	44.8 (32.4)	38.1 (15.1–60.5)
RRM	511	2 (0.4)		
P/LP other^b^	61	0 (0)	NA	NA
VUS only^c^	40	1^e^ (2.5)	5.7 (NA)	5.7 (NA)
Negative^d^	410	1^e^ (0.2)	6.7 (NA)	6.7 (NA)
RRSO	516	2 (0.4)		
Not yet recommended age^a^	4	0 (0)	NA	NA
P/LP other^b^	57	0 (0)	NA	NA
VUS only^c^	41	1 (2.4)	47.9 (NA)	47.9 (NA)
Negative^d^	414	1 (0.2)	43.3 (NA)	43.3 (NA)

Abbreviations: MRI, magnetic resonance imaging; P/LP, pathogenic/likely pathogenic; RRM, risk‐reducing mastectomy; RRSO, risk‐reducing oophorectomy; VUS, variant of uncertain significance.

^a^ Individuals with P/LP variant in high‐risk gene, intact organ(s), but had not reached recommended age for procedure.

^b^ Individuals with P/LP variant in other gene and intact organ(s).

^c^ Individuals with only VUS results and intact organ(s).

^d^ Individuals with negative results and intact organ(s).

^e^ *N* = 1 breast cancer survivor.

^f^ *N* = 3 breast cancer survivors.

**TABLE 5:** Use of one or more risk management strategy after results disclosure in those who did not receive actionable recommendations from CHARM study genetic counselors, by patient and system factors.

	*n* used at least one risk management strategy/*n* without any recommendations	Uptake (%)	*p* value
Study site			
KPNW	115/483	23.8	0.217
DH	36/186	19.4	
Access barriers^a^			
Yes	109/471	23.1	0.586
No	42/198	21.2	
Personal breast, ovarian, or colorectal cancer history			
Yes	9/27	33.3	0.172
No	142/642	22.1	
Knowledge of family history or structure			
Limited knowledge	17/146	11.6	**<0.0001**
Known family history	134/523	25.6	

*Note*: The bold front indicates *p*‐values with statistical significance. Abbreviations: DH, Denver Health; KPNW, Kaiser Permanente Northwest.

^a^ Any of the following: (1) self‐reported Hispanic or Latino ethnicity and/or a race other than White, (2) reside in a medically underserved area (>20% of households are below the poverty level and/or > 20% have less than a high school education) determined using geocoded census tract information, (3) completed any study survey or risk assessment tool in Spanish, (4) had less than high school education, (5) had income <200% federal poverty level or Medicaid insurance, or (6) were uninsured.

## 4 DISCUSSION

This study provides novel information about risk management behavior following cancer genetic testing through a health system‐embedded program designed to increase testing's reach to populations with historical barriers to access. Specifically, we found that delivering guideline‐indicated genetic testing through CHARM allowed high risk individuals in two health systems to pursue recommended cancer screening and/or risk reduction without prompting overuse of prophylactic surgery or supplemental screening among patients receiving negative or uncertain test results.

Despite the unique time period in which follow‐up occurred (early COVID pandemic), our results align with prior research and suggest that risk management use following implementation of health system‐embedded genetic testing programs may be similar in testing programs serving populations with and without access barriers.^18^, ^20^, ^32^, ^33^, ^34^, ^35^ Specifically, Geisinger's MyCode Community Health Initiative,^18^ which screened healthy patients for HBOC, LS, and familial hypercholesterolemia (FH) reported high post‐disclosure uptake of risk management for HBOC (72%, 82/114 had at least one recommended procedure) and moderate for LS (40%, 19/48 had at least one recommended procedure). Of note, individuals in this cohort were older than CHARM participants (median age of 63 years), followed for a longer period of time (median follow‐up 24 months), and almost exclusively non‐Hispanic White. The Healthy Nevada Project,^32^ another population genomic screening effort, reported that in the first year after testing 50% (29/58) of eligible patients had a mammogram or breast MRI. Like the MyCode cohort, these individuals were also older (mean age 53 years) and almost exclusively non‐Hispanic White, though this initiative was not fully embedded into a health system. We also did not observe high rates of risk management use among individuals who did not receive actionable recommendations from CHARM study genetic counselors. Only four individuals with negative or uncertain test results pursued risk reducing surgery during follow‐up, and two of these were cancer survivors. In contrast to individuals who received a new diagnosis of inherited breast cancer susceptibility, the use of breast MRI was rare among those who did not receive actionable recommendations and the majority of them who had mammograms and/or colonoscopies were over age 40 or 45 years, respectively. These findings are consistent with other work examining patient actions after clinical exome sequencing^19^, ^21^, ^36^ and suggest that overtreatment following cancer genetic testing is likely rarer than expected based on prior vignette and case studies.^37^, ^38^

Our findings offer a number of useful lessons for health care systems interested in implementing programs to provide clinically‐indicated cancer genetic testing to their patients. First, initiatives to equitably scale‐up risk assessment and genetic testing, like CHARM, can facilitate proactive risk management use. Many participants who received actionable risk management recommendations from study genetic counselors acted on at least one during study follow‐up, while those who did not were typically young women considering serious prophylactic surgeries with lifelong side‐effects. Given that results disclosure took place early during the COVID‐19 pandemic, observed screening engagement, particularly with breast MRI and colonoscopy, is quite promising. Second, implementing programs that specifically consider known barriers to accessing cancer genetic services, like CHARM did, can broaden genomic medicine's reach and narrow disparities in test access and subsequent risk management use. Specifically, we observed high engagement with risk management among participants belonging to sociodemographic groups with known access barriers and across two study sites—including a network of federally qualified health centers. Finally, though implementing a program like CHARM requires upfront investment to screen, counsel, and test patients, it is unlikely to put significant strain on pathways for delivering risk management that health systems already have in place. Of 680 participants included in our cohort, which represents 1753 who participated in risk assessment, only 23 had a P/LP variant in a well‐established colorectal, breast, and/or ovarian cancer gene, and only 11 received an actionable risk management recommendation. For health systems particularly concerned with meeting increased demand for risk management, rolling out genetic testing programs in stages or waves can provide an opportunity to proactively control and plan for increased patient volume.

Our study has several limitations. Few individuals received actionable risk management recommendations and those who did were homogeneous. These factors limit generalizability and constrained our ability to conduct analyses examining patient and health‐system factors associated with risk management use. We also had variable follow‐up times and short follow‐up duration for some participants, which impacted our likelihood of observing risk management across individuals. Many of the participants who were recommended to discuss or consider prophylactic surgery were much younger than the ages at which most women have been found to pursue these surgeries (mid‐forties).^39^ Therefore, the degree of engagement with risk management we observed should be confirmed in additional studies. To understand post‐testing behavior and its impact on health outcomes there is particular a need for studies that use alternative designs with longer follow‐up periods, more patients, and consider more actionable risk management interventions (i.e., clinical breast exam, chemoprevention).

Notably, we did not have sufficiently detailed clinical and family history data to determine if CHARM participants who did not receive actionable recommendations from study genetic counselors should otherwise consider enhanced risk management based on their lifetime breast cancer risk and/or specific family cancer history. Risk management recommendations made by study genetic counselors could also have differed from recommendations participants received before or after the study as part of usual clinical care. Adjudicating risk management adherence on an individual level would have required detailed chart review to abstract personalized recommendations, which was not possible given the size of our cohort. Therefore, we note that using individual's genetic test results, age, and surgical history alone, to define recommended care per NCCN guidelines was feasible, but may have led us to misclassify individual care recommendations. Finally, though costs have dramatically decreased and financial assistance programs are available, cancer genetic testing often has out of pocket costs, limiting the generalizability of engagement with free testing through CHARM to what may be expected if similar programs were implemented in usual care.

Despite these challenges, our study has many strengths. KPNW and DH's robust clinical and administrative data systems allowed for high‐quality capture of risk management procedures in stable, well‐defined populations, strengthening confidence in our results. We also successfully engaged and retained participants belonging to groups that traditionally experience barriers in accessing cancer genetic testing, providing important data on risk management uptake following testing in previously unstudied populations. Seventy percent of CHARM participants had low income, low literacy, and/or belonged to other marginalized groups with poor access to cancer genetic services. Our experience indicates that partnering with safety‐net health systems to conduct genomics research can be mutually beneficial, as it provides access to recommended care and increases genomics capacity, while increasing representation of individuals from diverse backgrounds and improving generalizability of study results. Finally, we provide important data on the likelihood of “overtreatment” following cancer genetic testing in a large number of individuals who were clinically eligible for genetic testing, but received negative or uncertain results. Previous research has focused largely on individuals with positive test results, overlooking the concerns surrounding anxiety or clinician mismanagement in those who tested negative or received VUS but were clinically suitable for genetic testing. This group comprises the majority of individuals who undergo genetic testing, raising important questions that warrant further investigation.

In conclusion, CHARM's experience can inform the integration of guideline‐recommended risk assessment and cancer genetic testing into clinical care. Findings from this study provide initial evidence that implementing approaches to delivering genetic services that consider traditional access barriers can provide broad benefits and potentially narrow disparities by allowing individuals to understand and act to reduce their personal cancer risk following testing.

## AUTHOR CONTRIBUTIONS

**Boya Guo:** Conceptualization (equal); data curation (lead); formal analysis (lead); investigation (lead); methodology (equal); visualization (equal); writing – original draft (equal); writing – review and editing (equal). **Sarah Knerr:** Conceptualization (equal); data curation (equal); investigation (equal); methodology (equal); project administration (equal); supervision (equal); writing – review and editing (equal). **Tia L. Kauffman:** Conceptualization (equal); project administration (equal); writing – review and editing (equal). **Kathleen F. Mittendorf:** Conceptualization (equal); methodology (equal); writing – review and editing (equal). **Erin M Keast:** Conceptualization (equal); data curation (equal); methodology (equal); writing – review and editing (equal). **Marian J. Gilmore:** Conceptualization (equal); methodology (equal); writing – review and editing (equal). **Heather Spencer Feigelson:** Conceptualization (equal); methodology (equal); writing – review and editing (equal). **Frances L Lynch:** Conceptualization (equal); methodology (equal); writing – review and editing (equal). **Kristin R Muessig:** Conceptualization (equal); methodology (equal); project administration (equal); supervision (equal); writing – review and editing (equal). **Sonia Okuyama:** Conceptualization (equal); data curation (equal); methodology (equal); writing – review and editing (equal). **Jamilyn M Zepp:** Methodology (equal); writing – review and editing (equal). **David L Veenstra:** Methodology (equal); writing – review and editing (equal). **Li Hsu:** Methodology (equal); writing – review and editing (equal). **Amanda I Phipps:** Methodology (equal); writing – review and editing (equal). **Sara Lindström:** Methodology (equal); supervision (equal); writing – review and editing (equal). **Michael C Leo:** Conceptualization (equal); funding acquisition (equal); methodology (equal); writing – review and editing (equal). **Katrina A.B. Goddard:** Conceptualization (equal); funding acquisition (equal); methodology (equal); project administration (equal); supervision (equal); writing – review and editing (equal). **Benjamin S. Wilfond:** Conceptualization (equal); funding acquisition (equal); methodology (equal); project administration (equal); supervision (equal); writing – review and editing (equal). **Beth Devine:** Conceptualization (equal); investigation (equal); methodology (equal); project administration (equal); supervision (equal); writing – review and editing (equal).

## FUNDING INFORMATION

This work was funded as part of the Clinical Sequencing Evidence‐Generating Research (CSER) consortium funded by the National Human Genome Research Institute with co‐funding from the National Institute on Minority Health and Health Disparities (NIMHD) and the National Cancer Institute (NCI). The CSER consortium represents a diverse collection of projects investigating the application of genome‐scale sequencing in different clinical settings including pediatric and adult subspecialties, germline diagnostic testing and tumor sequencing, and specialty and primary care. This work was supported by a grant from the National Human Genome Research Institute (U01HG007292; MPIs: Wilfond, Goddard, Leo), with additional support from U24HG007307 (Coordinating Center). The content is solely the responsibility of the authors and does not necessarily represent the official views of the National Institutes of Health.

## CONFLICT OF INTEREST STATEMENT

The authors declare no conflicts of interest.

## ETHICS STATEMENT

The KPNW IRB approved this study, and all collaborating IRBs ceded to KPNW except Dana Farber Cancer Institute and Columbia University, who approved the study separately.

## Supporting information


Table S1.

## Data Availability

The datasets generated and/or analyzed during the current study are not publicly available due to privacy and ethical restrictions but de‐identified datasets will be made available on the AnVIL platform (https://anvil.terra.bio/) and upon request.
