# Supplementary material for: Risk management actions following genetic testing in the Cancer Health Assessments Reaching Many (CHARM) Study: A prospective cohort study
Source: Cancer Med. 2023 Aug 30;12(18):19112–25. doi: 10.1002/cam4.6485 (PMC10557878; doi:10.1002/cam4.6485)
Supplement: Supplementary file 1 — Table S1. [file CAM4-12-19112-s001.docx]

**Supplementary Table 1**. Recommendations and test results of CHARM participants with a pathogenic and likely pathogenic (P/LP) variant in hereditary colorectal, breast and/or ovarian cancer related genes.

| **Gene** | **No. of individuals N (%)** | **CHARM study counselor recommendations** **for asymptomatic and unaffected individuals** | | |
| --- | --- | --- | --- | --- |
|  | **N=23** | **Breast cancer** | **Ovarian cancer** | **Colorectal cancer** |
| *APC* | 1 (4.3) | - | - | Screening: Annual colonoscopy every 12 months beginning at age 10-15 y |
| *ATM* | 2 (8.7) | Screening: Annual mammogram ages 40-75 y; Consider annual MRI ages 40-75 y |  |  |
| *BRCA1*  *BRCA2* | 2 (8.7)  4 (17.4) | Screening: Annual MRI ages 25-29 y; Annual mammogram and MRI ages 30-75 y  Risk reduction: Discuss option of RRM | Risk reduction: Recommend RRSO at ages 35-45 y | - |
| *BRIP1* | 1 (4.3) | - | Risk reduction: Recommend RRSO at ages 45–50 y | - |
| *CHEK2* | 6 (26.1) | Screening: Annual mammogram ages 40-75; Consider annual MRI ages 40-75 | - | Screening: Colonoscopy every 5 y, beginning at age 40 or 10 y prior to age of first-degree relative’s colorectal cancer diagnosis |
| *MSH2* | 1 (4.3) | - |  | Screening: Colonoscopy at age 20-25 y and repeat every 1-2 y |
| *MSH6* | 2 (8.7) | - |  | Screening: Colonoscopy at age 30–35 y and repeat every 1–2 y |
| *PALB2* | 1 (4.3) | Screening: Annual mammogram and annual MRI ages 30-75 y  Risk reduction: Discuss option of RRM | Risk reduction: Recommend RRSO at ages >45 y | - |
| *PMS2* | 2 (8.7) | - |  | Screening: Colonoscopy at age 30–35 y and repeat every 1–3 y |
| *RAD51C* | 1 (4.3) | Screening: Annual mammogram ages 30-75 y; Consider annual MRI ages 40-75 y | Risk reduction: Recommend RRSO at ages 45–50 y | - |

Abbreviations: MRI (magnetic resonance imaging), RRM (risk-reducing mastectomy), and RRSO (risk-reducing salpingo-oophorectomy).

**Supplementary Table 2**. Flow chart of eligible individuals after test result disclosure.

| **Exclusion Criteria** | **Sample size**  **N (%)** |
| --- | --- |
| **Breast cancer screening/RRM** | |
| **Step 1.** CHARM participants included in analysis | 680 (100.0) |
| **Step 2.** Exclude those who were not assigned as female sex at birth (N=138) | 542 (79.7) |
| **Step 3.** Exclude those without intact breasts before the test result returned date (N=26) | 516 (75.9) |
| **Eligible sample size** | **516** |
| **RRSO** |  |
| **Step 1.** CHARM participants included in analysis | 680 (100.0) |
| **Step 2.** Exclude those who were not assigned as female sex at birth (N=138) | 542 (79.7) |
| **Step 3.** Exclude those without intact ovaries before the test result returned date (N=23) | 519 (76.3) |
| **Eligible sample size** | **519** |
| **Colorectal cancer screening** | |
| **Step 1.** CHARM participants included in analysis | 680 (100.0) |
| **Step 2. Exclude those without intact colon before the test result returned date (N=9)** | 671 (98.7) |
| **Eligible sample size** | **671** |

Abbreviations: RRM (risk-reducing mastectomy), RRSO (risk-reducing salpingo-oophorectomy).

**Supplementary Table 3**. Procedure codes of risk management options.

| **Code type** | **Code** |
| --- | --- |
| **Mammography** | |
| CPT | 77051, 77052, 77055, 77056, 77057, 77061, 77063, 77065, 77066, 77067 |
| HCPCS | G0202, G0204, G0206 |
| ICD9PCS | 87.36, 87.37, V76.11, V76.12 |
| **MRI** |  |
| CPT | 0159T, 76093, 76094, 77047, 77049, 77058, 77059 |
| HCPCS | C8903-05, C8906-08 |
| **Colonoscopy** |  |
| CPT | 0066T, 45378, 45380, 45381, 45382, 45383, 45384, 45385, 45386, 45388, 45390, 74261, 44388, 44389, 44390, 44391, 44392, 44393, 44394, 44397, 44401, 44402, 44403, 44404, 44405, 44406, 44407, 44408, 45379, 45387, 45389, 45391, 45392, 45393, 45398, 74262 |
| HCPCS | G0105, G0121 |
| ICD9 | 45.22, 45.23, 45.25, 45.42, 45.43 |
| **Bilateral mastectomy** | |
| ICD10 | 0HTV0ZZ |
| ICD9 | 85.35; 85.36; 85.42; 85.44; 85.46; 85.48 |
| CPT | 19303 with 50 modifier code |
| **Unilateral mastectomy** | |
| ICD10 | 0HTT0ZZ, 0HTU0ZZ |
| ICD9 | 85.33, 85.34, 85.41, 85.43, 85.45, 85.47 |
| CPT | 19180, 19182, 19200, 19303 without 50 modifier code, 19305, 19307, 19220, 19240, 19304, 19306, 19304, 19306 |
| **Bilateral salplingo-oophorectomy** | |
| ICD10 | 0UT20ZZ; 0UT24ZZ; 0UT27ZZ; 0UT2FZZ; Z40.04 |
| ICD9 | 65.51; 65.53; 65.61; 65.63; V50.42 |
| CPT | 58950; 58951; 58952; 58953; 58954; 58956 |

Abbreviation: MRI (magnetic resonance imaging)

**Supplementary Table 4**. ICD-O codes of hereditary breast, ovarian, and colorectal cancers.

| **Cancer** | **ICD-O Code** |
| --- | --- |
| **Breast cancer** | C50.X |
|  |  |
| **Colorectal cancer (excludes anus)** | C18.X, excluding C18.1 (colon) |
|  | C19.9 (rectosigmoid junction) |
|  | C20.9 (rectum, not otherwise specified) |
|  |  |
| **Ovarian (includes tubal and peritoneal)** | C48.X (Peritoneal) |
|  | C56.9 (Ovary) |
|  | C57.0 (Fallopian tube) |
|  | C57.1 (Broad ligament) |
|  | C57.2 (Round ligament) |
|  | C57.3 (Parametrium) |
|  | C57.4 (Uterine adnexa) |

**Supplementary Table 5.** Pathogenic and likely pathogenic (P/LP) index test results in genes that do not have a NCCN screening and risk reducing surgery recommendation for hereditary colorectal, breast and/or ovarian cancer.

| **Gene** | **Number of individuals**  N=70^a^ | **Primary condition** |
| --- | --- | --- |
| *APOB* | 1 (1.4) | Familial hypercholesterolemia |
| *CFTR* | 19 (27.1) | Cystic fibrosis |
| *DSG2* | 1 (1.4) | Arrhythmogenic right ventricular dysplasia |
| *FANCM* | 1 (1.4) | Breast cancer^b^ |
| *G6PD* | 3 (4.3) | Hemolytic anemia |
| *GBA* | 2 (2.9) | Gaucher disease |
| *GJB2* | 16 (22.9) | Keratitis-ichthyosis-deafness syndrome |
| *HEXA* | 1 (1.4) | Infantile Tay-Sachs disease |
| *IDUA* | 1 (1.4) | Mucopolysaccharidosis |
| *LDLR* | 1 (1.4) | Familial hypercholesterolemia |
| *MUTYH heterozygotes* | 16 (22.9) | Colorectal cancer^c^ |
| *MYBPC3* | 2 (2.9) | Cardiomyopathy |
| *PTCH1* | 1 (1.4) | Non-syndromic holoprosencephaly |
| *RYR1* | 1 (1.4) | Malignant hyperthermia susceptibility |
| *SDHD* | 1 (1.4) | Gastrointestinal stromal tumor |
| *SERPINA1* | 1 (1.4) | Emphysema due to AAT deficiency |
| *SLC22A5* | 1 (1.4) | Primary carnitine deficiency |
| *SMPD1* | 1 (1.4) | Niemann-Pick disease |
| *TMEM127* | 1 (1.4) | Pheochromocytomas/paragangliomas |
| *TNNI3* | 1 (1.4) | Cardiomyopathy |
| *TSC1* | 1 (1.4) | Tuberous sclerosis complex |

^a^Numbers do not add up to 70 because 1 patient had P/LP in *CFTR* and *GBA*, 1 patient had P/LP in *GJB2* and *MUTYH*, and 1 had P/LP in *GJB2* and *SDHD.*

^b^A P/LP variant in *FANCM* has inconsistent evidence of increasing breast cancer risk, and is not recommended by NCCN guidelines to have breast cancer risk management.

^c^Colorectal cancer screening is only recommended on the basis of the finding of a single P/LP variant in *MUTYH* when there is a family history of colon cancer in a first-degree relative. Participants with this finding were also not considered as having NCCN screening recommendations for colon cancer.
